# *Bacillus cereus* CGMCC 1.60196: a promising bacterial inoculant isolated from biological soil crusts for maize growth enhancement

**DOI:** 10.3389/fmicb.2024.1461949

**Published:** 2024-09-09

**Authors:** Lina Zhao, Chenrui Kang, Shipeng Zhang, Linlin Cui, Shuaihua Xu, Yudong Wang, Yue Zhang, Shaobin Gu

**Affiliations:** ^1^College of Food and Bioengineering, Henan University of Science and Technology, Luoyang, China; ^2^Henan Engineering Research Center of Food Microbiology, Luoyang, China; ^3^National Demonstration Center for Experimental Food Processing and Safety Education, Luoyang, China

**Keywords:** *Bacillus cereus*, carbon degradation, cyanobacterial crusts, extracellular hydrolase activity, plant growth-promoting bacteria, response surface methodology

## Abstract

Soil microbial inoculants are widely recognized as an environmentally friendly strategy for promoting crop growth and increasing productivity. However, research on utilizing the microbial resources from desert biological soil crusts to enhance crop growth remains relatively unexplored. In the present work, a bacterial strain designated AC1-8 with high levels of amylase, protease, and cellulase activity was isolated from cyanobacterial crusts of the Tengger Desert and identified as *Bacillus cereus* (CGMCC 1.60196). The refinement of the fermentation parameters of *B. cereus* CGMCC 1.60196 determined that the most effective medium for biomass production was composed of 5 g/L glucose, 22 g/L yeast extract and 15 g/L MgSO_4_, and the optimal culture conditions were pH 6.0, temperature 37°C, inoculation quantity 3% and agitation speed 240 rpm. Furthermore, the utilization of *B. cereus* CGMCC 1.60196 has resulted in substantial improvements in various growth parameters of maize seedlings, including shoot length, shoot fresh and dry weights, root fresh and dry weights, and the contents of chlorophyll *a*, chlorophyll *b*, and total chlorophyll. The most pronounced growth promotion was observed at an application concentration of 1 × 10^9^ CFU/m^2^. These results suggest that the novel *B. cereus* strain, isolated from cyanobacterial crusts, can be regarded as an exemplary biological agent for soil improvement, capable of enhancing soil conditions, promoting crop cultivation and supporting food production.

## 1 Introduction

Crop cultivation represents a fundamental aspect of human existence, providing sustenance and economic stability. However, the ever-increasing global population and the subsequent demand for food have placed immense pressure on soil vitality and agricultural practices (Cordell et al., 2009; Aytenew, 2021). Traditional methods of crop cultivation, while effective, frequently depend on synthetic fertilizers that may result in environmental degradation and social burdens, including soil erosion and degradation, desertification, contamination of water resources, loss of biodiversity, and impacts on economic trends (Smeets and Faaij, 2010; Pahalvi et al., 2021). Consequently, there is a burgeoning interest in exploring alternative, eco-friendly approaches to augment soil fertility and enhance crop growth.

In the pursuit of sustainable agricultural practices, soil microbial inoculants are considered a beneficial agricultural strategy that can improve soil conditions and increase crop yield by promoting the absorption and utilization of mineral nutrients, while minimizing environmental impact (Sharma et al., 2016; de Vries et al., 2020; Devi et al., 2022). *Bacillus* species, spore-forming bacterium widely distributed in soil environments, are known for their secretion of diverse enzymes that facilitate nutrient cycling, exert a broad spectrum of positive influences on enriching soil available resources and promoting crop growth (Ongena and Jacques, 2007; Bhattacharyya et al., 2017; Vinodkumar et al., 2017; Zhang et al., 2023). Additionally, *Bacillus* spp., widely recognized as safe, has emerged as a robust organism capable of not only growing readily to very high densities but also remaining stable and easy to store subsequent to the preparation of bacterial agents, and it can swiftly recover, reproduce, and function effectively within a suitable environment (Kumar et al., 2011; Gu et al., 2015). Therefore, *Bacillus* spp. have become ideal candidates for biofertilizer formulations, owing to their characteristics and beneficial contributions to agricultural practices.

Biological soil crusts (BSCs), communities of cyanobacteria, lichens, mosses, lichens, fungi, archaea and bacteria that live in the uppermost few millimeters of soil, and make up 40% of the living cover of desert ecosystems (Belnap and Lange, 2003; Weber et al., 2016). As the early developmental stage of BSC development, the formation and development of cyanobacterial crusts are often accompanied by dramatic increases in soil fertility, which is crucial for the subsequent development and stability of the entire ecosystem (Li et al., 2004, 2006). It has been widely accepted for many years that Cyanobacteria play important roles in the improvement of soil fertility and stability during BSC development, and recent studies have further confirmed the contribution of bacterial species, which multiply more rapidly than Cyanobacteria in cyanobacterial crusts, to these processes (Pepe-Ranney et al., 2016; Park et al., 2017; Zhao et al., 2020).

*Bacillus* as the dominant genus (with the highest relative abundance) of cyanobacterial crusts (Liu et al., 2017a,b), has ecological functions such as carbon degradation (mainly the degradation of starch and cellulose), carbon fixation and ammonification. Guida et al. (2023) found that inoculation of cyanobacteria and/or bacteria, particularly strains such as *B. subtilis* and members of the *Azotobacter* genus, can lead to a substantial alteration in the soil hydrological processes. Mao et al. (2023) found that BSCs formed by co-inoculation of *Bacillus* XZM and microalgae remediate arsenic-contaminated soil in mining areas. Our previous study found that *B. tequilensis* CGMCC 17603 with high productivity of exopolysaccharide can effectively improve the ability of sand fixation and wind prevention (Zhao et al., 2019). Therefore, *Bacillus* in cyanobacterial crusts is expected to become a crucial microbial resource to promote the restoration of degraded soils.

In this work, our goals were (i) to isolate, screen and identify a *Bacillus* sp. with high amylase, protease and cellulase activities, (ii) to optimize the fermentation conditions of the isolate, and (iii) to assess the phytobeneficial effects of the strain on maize seedling growth. The results of this study provide promising microbial resource for the development of microbial fertilizers or soil amendments, which are essential to promote the improvement of farmland quality and the quality and yield of farmland crops.

## 2 Materials and methods

### 2.1 Isolation, screening and identification of candidate strain

In July 2021, cyanobacterial crusts were collected from the Shapotou revegetated area (37°32′N, 105°02′E), southeastern edge of the Tengger Desert, North Central China. The annual average temperature was 9.6°C, the extreme minimum temperature was −25.1°C, and the extreme maximum temperature was 38.1°C. Six samples were collected, placed inside a sterile plastic reservoir to form a composite sample, transported to the laboratory in a cooler packed with ice, and stored at 4°C until further processing. The isolation of *Bacillus* strains was carried out according to the method of our previous study (Zhao et al., 2019). To identify the candidate strain, the extracellular hydrolase activities of the isolates was further examined using the Oxford Cup method. Briefly, sterile agar medium was poured into sterile petri dishes (90 mm diameter), four Oxford cups were placed equidistantly on the surface of individual petri dish after medium solidify, 200 μL of fresh overnight culture was added to three cups whereas an equivalent volume of sterile saline was added to the other cup as comparison, and then the petri dishes were incubated at 37°C for 24 h before observing the hydrolysis zone. The agar mediums and hydrolysis zone observation method used for the determination of extracellular hydrolase activities were carried out as previously described (Gu et al., 2015). In addition, genetic identification was carried out by 16S rRNA sequencing according to Sun et al. (2023).

### 2.2 Optimization of fermentation medium and conditions of candidate strain

#### 2.2.1 Optimization of fermentation conditions of candidate strain

Fermentation conditions included pH (5.5, 6.0, 6.5, 7.0 and 8.0), temperature (34, 37, 40, and 43°C), inoculation quantity (2.0, 2.5, 3.0, 3.5 and 4.0%), and agitation speed (180, 200, 220, and 240 rpm) were optimized by changing one factor at a time and keeping all the other factors constant. The experiments were conducted in 250 mL Erlenmeyer flasks with 30 mL of medium for 24 h, and then the optical density in each case was monitored at 600 nm using a spectrophotometer.

#### 2.2.2 Optimization of fermentation medium of candidate strain

Optimal fermentation medium for biomass production, including carbon sources (glucose, sucrose, maltose and lactose), nitrogen sources [beef extract, yeast extract, urea and (NH_4_)_2_SO_4_], and inorganic salts (NaCl, MgSO_4_, K_2_HPO_4_ and NaH_2_PO_4_). The concentrations of the above carbon sources and inorganic salts were ranges from 10 to 30 g/L and 5 to 25 g/L, respectively. The concentrations of each nitrogen source ranged from G1 to G5: beef extract from 5 to 25 g/L, yeast extract from 10 to 30 g/L, urea from 2.5 to 12.5 g/L, and (NH_4_)_2_SO_4_ from 2.5 to 12.5 g/L. These parameters were tested individually while keeping other components of LB medium constant (10 g/L tryptone, 5 g/L yeast extract, 10 g/L NaCl, pH 7.0) to evaluate their impact on the biomass yield of the strain.

Response Surface Methodology (RSM), an empirical statistical modeling technique, can minimize the number of experiments required to evaluate several independent variables and their interactions (Ravikumar et al., 2007; Amenaghawon et al., 2013). The main carbon source (*A*), main nitrogen source (*B*) and main inorganic salt (*C*) were optimized based on a three-level (−1, 0 and + 1), three-variable Box–Behnken design (BBD). A total of 17 experiments (runs 1–17) were designed, including 5 central point experiments. Biomass was estimated as CFU/mL using 100 μL inoculum diluted in ten-fold steps. Design Expert software 8.0.6 (Stat-Ease Inc., Minneapolis, MN, United States) was used for regression of the experimental data. Analysis of variance (ANOVA) was used to determine the significance of the main and interaction effects of model terms.

### 2.3 Phytobeneficial effects of candidate strain on maize seedling growth

Three maize seeds (Xianyu 335) were planted in each pot with 2.5 kg of low-fertility soil containing 53.54 ± 12.96 mg/kg available nitrogen, 19.46 ± 4.28 mg/kg available phosphorus, and 130.95 ± 2.81 mg/kg available potassium, and then the inoculum of candidate strain was sprayed on the soil surface. The inoculum concentration was divided into three levels, 1 × 10^8^ CFU/m^2^ (T1), 1 × 10^9^ CFU/m^2^ (T2) and 1 × 10^10^ CFU/m^2^ (T3) respectively, and the control pot (T0) was planted with seeds that received the same volume of sterile water. Treatments were replicated three times and were completely randomized. All pots were maintained at 60% of soil water-holding capacity. Plants received a photoperiod of 16/8 h light/dark and temperatures of 24/18°C day/night. Observations were taken on the 49th day of treatment. Growth parameters such as chlorophyll content (Guo et al., 2022), height, wet weight, dry weight of the shoot and root (Liu et al., 2021) were measured and statistically analyzed.

### 2.4 Statistical analysis

ANOVA and Tukey’s honestly significant difference analysis were performed using SPSS for Windows, Version 16.0 (SPSS Inc., Chicago, IL, United States) to determine differences among various treatments for the crop growth parameters, provided that the data met the assumptions of normality of residuals and homoscedasticity. Mann–Whitney *U*-test was used to examine the differences among various treatments for data that failed to meet the assumptions of normality of residuals and homoscedasticity. Figures were generated using Origin 8.0 (Origin Lab Corp., Northampton, MA, United States).

## 3 Results and discussion

### 3.1 Screening, isolation and identification of candidate *Bacillus* strain

Six isolates, designated as *Bacillus* spp. AC1-6, AC1-8, AC3-2, AC3-4, AC3-5 and AC4-7, displayed amylase, cellulase and/or protease activity (Table 1). The observation of extracellular hydrolytic enzyme activities of isolated AC1-8 to starch (24.0 mm), cellulose (29.6 mm) and protein (24.6 mm) showed that the isolate may synergistically promote the decomposition of starch, cellulose and protein. Furthermore, the application of the strain in soil environment has the potential to promote plant nutrient absorption and enhance soil nutrient cycling (Zhao et al., 2020; Miao et al., 2021).

**Table 1:** Hydrolytic activity of the different *Bacillus* strains to starch, cellulose and protein.

Strains	Zone diameter (mm)		
	Amylase	Cellulase	Protease
AC1-6	20.3	0	23.0
AC1-8	24.0	29.6	24.6
AC3-2	16.2	24.9	23.2
AC3-4	19.7	0	22.7
AC3-5	14.7	0	23.8
AC4-7	18.6	17.0	18.6

**Figure 1:**
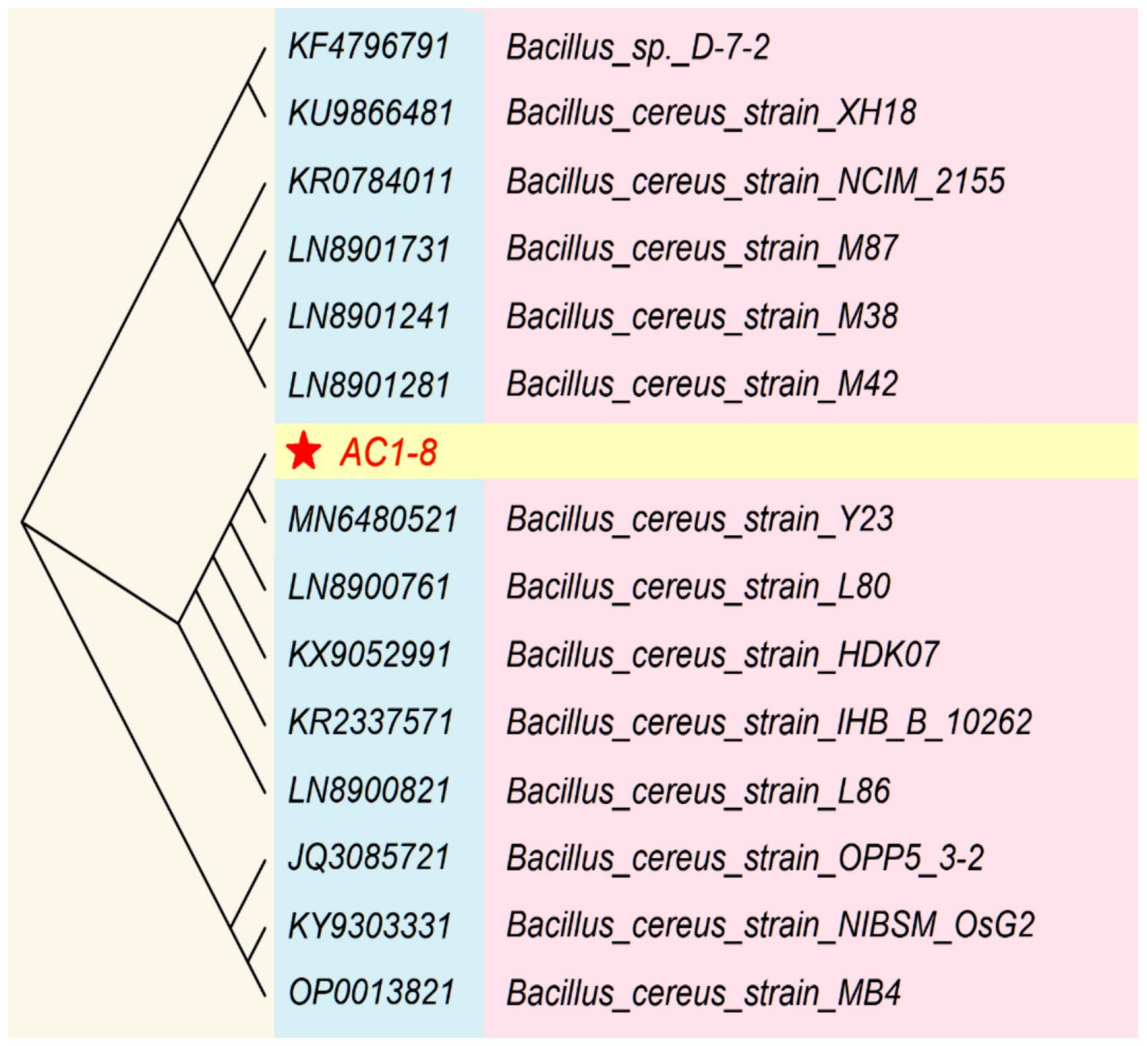
Phylogenetic analysis based on 16S rRNA gene sequences available from the National Center for Biotechnology Information.

The identification of the isolated AC1-8 strain was based on both morphological and molecular methods. Strain AC1-8 was observed to be Gram-positive, spore-forming and rod-shaped. Colonies on LB plate was circular, smooth, yellowish white, and approximately 3 mm in diameter after incubation for 24 h at 37°C. These characteristics are similar to those of *Bacillus* species. Moreover, the 16S rDNA sequences of AC1-8 were aligned using the NCBI-BLAST database, indicating that the genetic sequence was 98% homologous to that of the *Bacillus cereus* Y23 strain (GenBank accession number: MN6480521) (Figure 1). Therefore, AC1-8 was identified as *B. cereus*, and has been deposited at China General Microbiological Culture Collection Center (CGMCC) under deposition number of CGMCC 1.60196.

### 3.2 High concentration culture of *Bacillus cereus* CGMCC 1.60196

To realize the popularization and application of *B. cereus* CGMCC 1.60196 in the field of soil improvement and plant growth promotion, high biomass yield and the low cost of this production process are the key problems to be solved. Temperature, pH, inoculation quantity and agitation speed are critical parameters that significantly influence the production of bacterial biomass in fermentation processes (Freitas et al., 2010). Similarly, the production of bacterial biomass was also largely influenced by the medium compositions, such as the C source, the N source and the inorganic salt (Luang-In et al., 2018). By optimizing these parameters, it is possible to enhance the efficiency and productivity of the fermentation process, leading to increased bacterial biomass production (Rao et al., 2007; Kumar et al., 2011). Therefore, our study optimized the culture conditions and medium compositions of *B. cereus* CGMCC 1.60196, as these were the most important factors affecting the production of bacterial biomass and metabolites.

**Figure 2:**
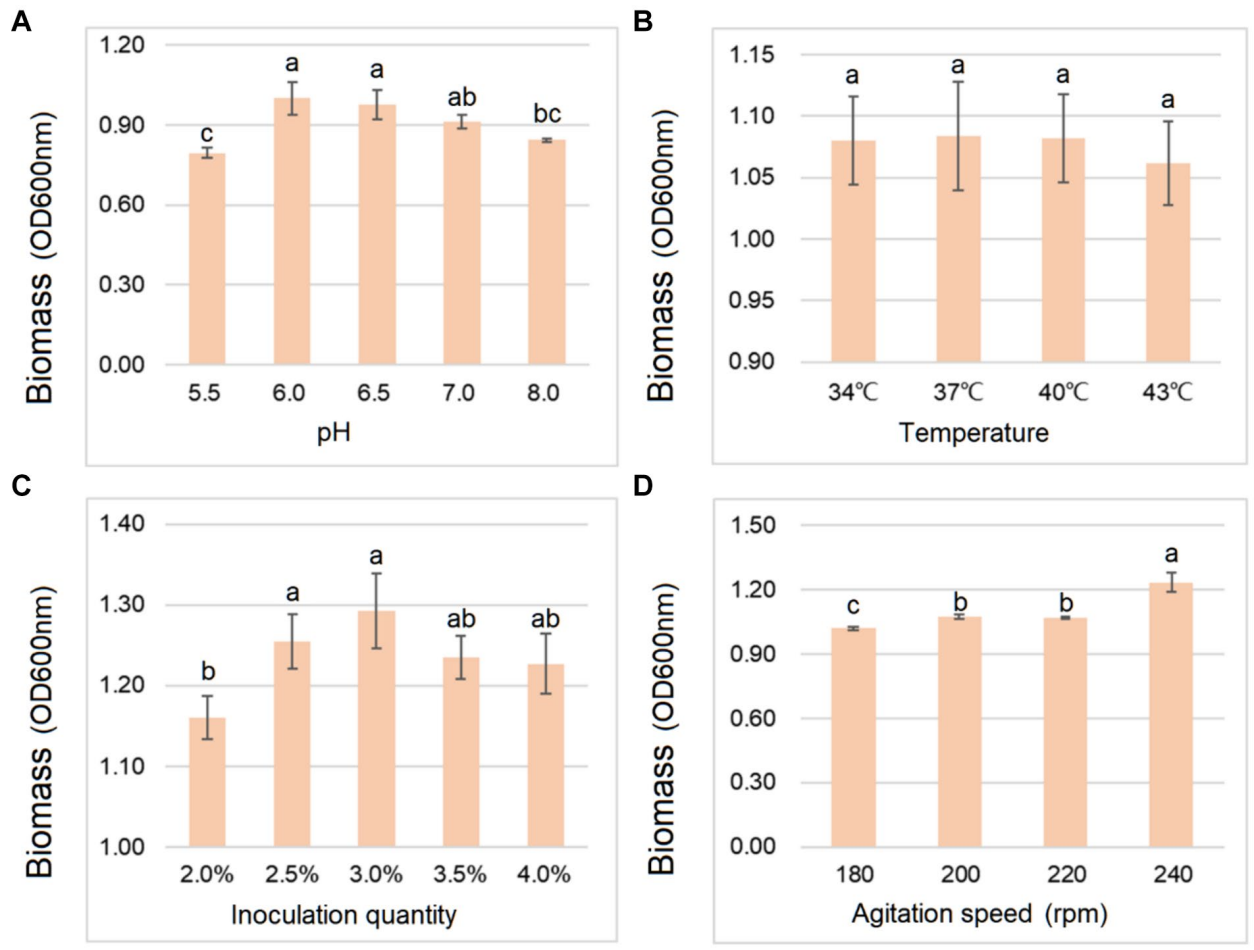
Effects of different gradients of pH **(A)**, temperature **(B)**, inoculation quantity **(C)**, and agitation speed **(D)** on the biomass yield of *B. cereus* AC1-8. Different lowercase letters represent significant differences between variables (*p* < 0.05). Error bars represent standard deviation of the mean (*n* = 3).

**Figure 3:**
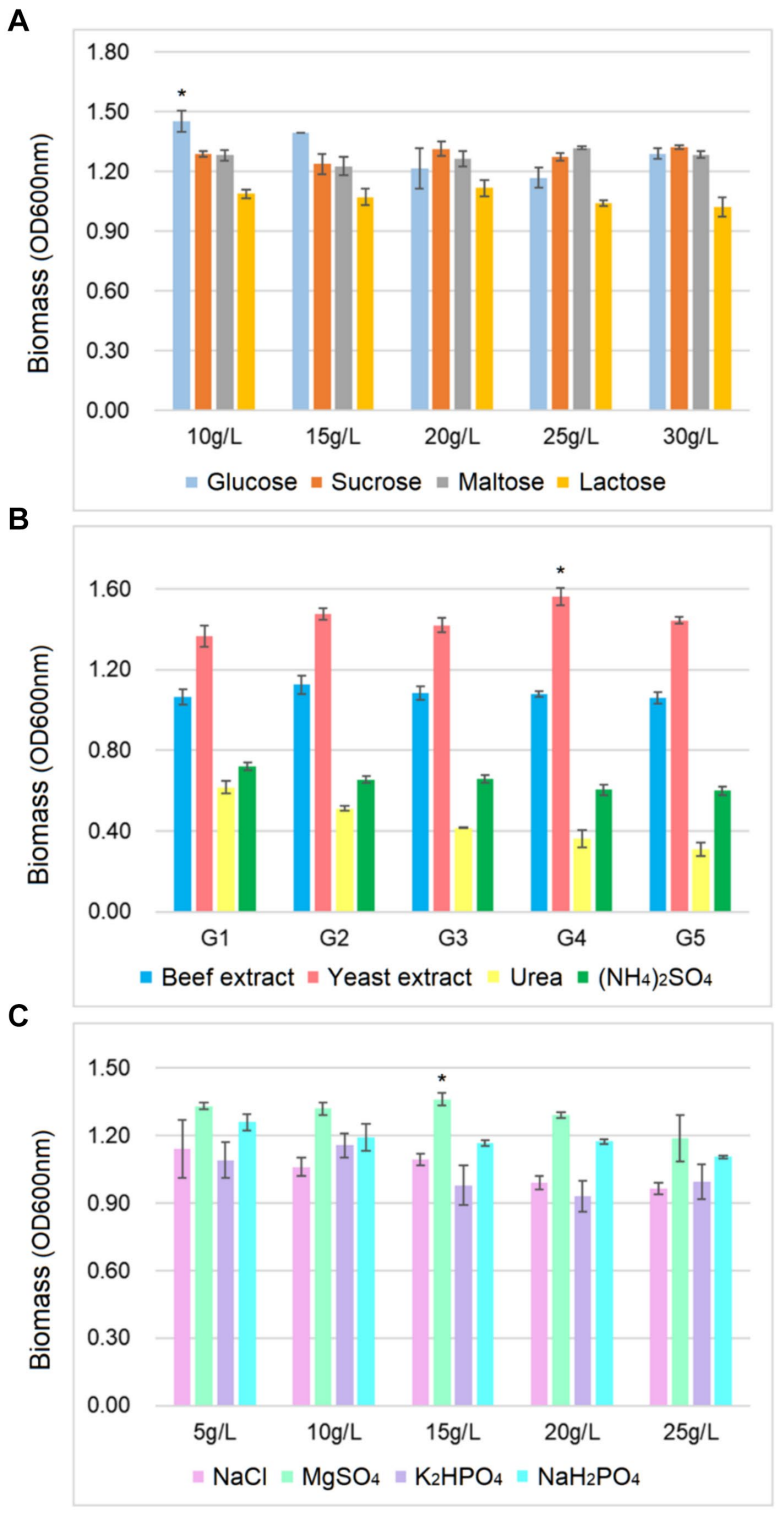
Effects of different concentrations of carbon source **(A)**, nitrogen source **(B)** and inorganic salt **(C)** on the biomass yield of *B. cereus* AC1-8. ∗ represent a significant difference between variables (*p* < 0.05). Error bars represent standard deviation of the mean (*n* = 3).

As shown in Figure 2, the biomass of *B. cereus* CGMCC 1.60196 was higher when pH, inoculation quantity and agitation speed were controlled at 6.0, 3% and 240 rpm, respectively. Considering the energy consumption of fermentation, the optimal temperature was set at 37°C. Additionally, the optimal C source, N source, and inorganic salt of *B. cereus* CGMCC 1.60196 were selected from multi-nutrient and multi-level, which were 10 g/L glucose, 25 g/L yeast extract, and 15 g/L MgSO_4_, respectively (*p* < 0.05; Figure 3). Consistent with previous studies, the optimal levels of key factors can be successfully screened through single-factor experiments, and the yield of target products was increased by multiple times (Mcdaniel and Ankenman, 2000; Ahmed et al., 2019; Saha and Mazumdar, 2019). However, these experiments do not take into account the possible interactions between the factors, thereby resulting in an inability to obtain an optimal strategy.

RSM, as an effective mathematical and statistical tool, was selected to improve the biomass production of *B. cereus* CGMCC 1.60196 by studying all factors involved in experimental analysis and their possible interactions to optimize the fermentation process (Ramić et al., 2015; Hippolyte et al., 2018; Araya-Farias et al., 2019). In this work, 17-run experiments were performed via BBD based on three variables in three levels to obtain the maximum biomass production of *B. cereus* CGMCC 1.60196 (Tables 2, 3). Run 8 showed the maximum biomass production when the medium compositions were set to 5 g/L glucose, 20 g/L yeast extract and 15 g/L MgSO _4_ (Table 3). Multiple regression analysis on the actual responses, established first-order polynomial equation to explain the biomass production of *B. cereus* CGMCC 1.60196 as following:

**Table 2:** Independent variables and levels used for Box–Behnken design.

Independent variables	Symbol coded	Levels		
		−1	0	+1
Glucose	*A*	5	10	15
Yeast extract	*B*	20	25	30
MgSO_4_	*C*	10	15	20

**Table 3:** Box–Behnken design matrix for the three independent variables and their corresponding experimental observed responses.

Run	*A*	*B*	*C*	Biomass (10^10^CFU/ml)
1	10	25	15	1.67
2	10	30	10	1.55
3	15	20	15	1.65
4	10	25	15	1.67
5	10	25	15	1.70
6	10	30	20	1.49
7	5	25	20	1.80
8	5	20	15	1.87
9	10	20	20	1.65
10	5	30	15	1.72
11	10	25	15	1.72
12	15	25	10	1.75
13	5	25	10	1.79
14	10	25	15	1.68
15	10	20	10	1.66
16	15	30	15	1.67
17	15	25	20	1.70

*A: glucose; B: yeast extract; C: MgSO4.*


(1) $$\begin{array}{l} Y=+0.714475-0.125705A+0.109625B+0.057730C\\ +0.001610\mathrm{AB}-0.000620\;\mathrm{AC}-0.000460\;BC\\ +0.004219A^{2}-0.002581B^{2}-0.001431C^{2} \end{array}$$

Based on the results of ANOVA, the significance value of the model (*F*-value = 14.00; *p*-value = 0.00011), and a non-significant value lacking a fit (*p*-value = 0.1018) indicated that model provided here valid (Table 4; Amouzgar et al., 2010). The determination of the squared regression coefficient (*R*^2^) for biomass production was 0.9474 meaning that the data variability could be interpreted very well by the models. The value of the adjusted *R*^2^ (0.8797) also supported a good fit between the regression model and to the data (Table 4). In addition, linear terms (*A* and *B*), quadratic terms (*A*^2^, *B*^2^ and *C*^2^) and interaction term (*AB*) were significant (*p* < 0.05) whereas others were not significant model terms (Table 4), suggesting that glucose and yeast extract have a significant effect on the biomass production of *B. cereus* CGMCC 1.60196. The maximum predicted value of biomass production was 1.87 × 10^10^ CFU/mL when the parameters were set to glucose 5 g/L, yeast extract 22 g/L, and MgSO_4_ 15 g/L. Under the recommended optimum conditions, the biomass production of *B. cereus* CGMCC 1.60196 was 1.91 × 10^10^ ± 2.31 × 10^7^ CFU/mL, which was in close agreement with the predicted value.

**Table 4:** ANOVA analysis and statistical parameters of the fitted quadratic polynomial model for the optimization of medium compositions.

Source	Sum of squares	Degrees of freedom	Mean square	*F*-value	*p*-value	
Model	0.1184	9	0.0132	14.00	0.0011	Significance
*A*	0.0215	1	0.0215	22.91	0.0020	**
*B*	0.0209	1	0.0209	22.25	0.0022	**
*C*	0.0017	1	0.0017	1.79	0.2227	ns
*AB*	0.0065	1	0.0065	6.90	0.0341	*
*AC*	0.0010	1	0.0010	1.02	0.3455	ns
*BC*	0.0005	1	0.0005	0.56	0.4775	ns
*A*^2^	0.0468	1	0.0468	49.85	0.0002	**
*B*^2^	0.0175	1	0.0175	18.66	0.0035	**
*C*^2^	0.0054	1	0.0054	5.74	0.0478	*
Residuals	0.0066	7	0.0009			
Lack of fit	0.0050	3	0.0017	4.14	0.1018	Not significance
Pure error	0.0016	4	0.0004			
Cor total	0.1249	16				
*R*^2^ = 0.9474						
*Adjusted R*^2^ = 0.8797						

*A: glucose; B: yeast extract; C: MgSO4.* *, significant (*p* < 0.05); **, very significant (*p* < 0.01); ns, not significant (*p* > 0.05).

### 3.3 Effect of *Bacillus cereus* CGMCC 1.60196 on seedling growth

Soil microbial inoculants, increasingly adopted to enhance crop productivity, face significant challenges in screening, preserving functional properties, and ensuring efficient application in low-fertility soils (Singh, 2017; Kaminsky et al., 2019). In this study, *B. cereus* CGMCC 1.60196, which was isolated from cyanobacterial crusts and showed a perfect potential to improve soil nutrient conditions, was chosen in order to highlight its effect on seedling growth in low fertility soil. As shown in Table 5, the shoot length, shoot fresh weight, shoot dry weight, root fresh weight, root dry weight, and contents of total chlorophyll, chlorophyll *a* and chlorophyll *b* of the T2 treatment group were significantly higher than those of other treatment groups, indicating that *B. cereus* CGMCC 1.60196 exhibited good physiological growth-promoting characteristics on maize seedlings. Consistent with previous researches, plants inoculated with *B. megaterium*, *B. aryabhattai* and/or *B. mesonae* significantly enhance tomato growth and chlorophyll content (Fan et al., 2016; Yoo et al., 2019).

**Table 5:** Effect of *B. cereus* CGMCC 1.60196 on the maize seedling growth.

Treatment	Shoot			Root		Leaf		
	Length (cm)	Fresh weight (g)	Dry weight (g)	Fresh weight (g)	Dry weight (g)	Total chlorophyll (mg/g)	Chlorophyll *a* (mg/g)	Chlorophyll *b* (mg/g)
T0	73.03 ± 1.91 b	24.39 ± 0.69 b	3.06 ± 0.32 c	7.45 ± 0.03 d	1.54 ± 0.06 b	11.82 ± 0.47 b	9.47 ± 0.28 b	2.35 ± 0.30 b
T1	74.30 ± 3.11 b	25.89 ± 1.33 b	3.52 ± 0.12 b	8.40 ± 0.21 c	1.67 ± 0.32 b	12.63 ± 0.36 b	10.32 ± 0.24 b	2.31 ± 0.16 b
T2	81.00 ± 1.47 a	36.91 ± 2.17 a	4.69 ± 0.18 a	15.09 ± 0.35 a	3.45 ± 0.36 a	17.78 ± 3.61 a	14.15 ± 2.75 a	3.63 ± 0.86 a
T3	63.67 ± 2.83 c	27.13 ± 2.30 b	3.80 ± 0.15 b	10.30 ± 0.15 b	1.71 ± 0.17 b	15.58 ± 0.39 ab	12.42 ± 0.22 ab	3.16 ± 0.30 ab

Lowercase letters indicate significant differences between treatments (*p* < 0.05). Data shown represent means ± standard deviation (*n* = 3).

Moreover, some *Bacillus* spp. have been identified as plant growth-promoting bacteria that can stimulate plant growth through direct or indirect mechanisms (Podile and Kishore, 2006). For example, by increasing the bioavailability of mineral nutrients (as demonstrated in this study) (Samreen et al., 2019; Cardoso et al., 2021), or by providing exopolysaccharides, amino acids, and other nutritional factors (Zhao et al., 2019; Khan et al., 2021), or by synthesizing plant growth regulating compounds such as gibberellin and indole acetic acid (Gutiérrez-Mañero et al., 2001; Khan et al., 2021). *B. cereus* CGMCC 1.60196 has excellent extracellular hydrolytic enzyme activities (Table 3), providing maize seedlings with greater potential for nutrient uptake and transport, resulting in greater growth in maize seedlings (Zhao et al., 2020; Zhou et al., 2022). Microbial inoculants with agriculture-related traits such as nutrient facilitation are essential for effectively promoting plant growth in low-fertility soils (Jiang et al., 2023). In addition to enhancing nutrient absorption, whether *B. cereus* CGMCC 1.60196 produces hormones or other secretions to promote the growth of maize seedlings is still unknown.

## 4 Conclusion

In summary, this study provided a new strain of *B. cereus* CGMCC 1.60196 with high amylase, protease and cellulase activities, realized its high density fermentation, and revealed its promoting effects on the growth of maize seedlings. This discovery not only broadens our understanding of microbial resources in extreme environments such as desert BSCs, but also offers a valuable, eco-friendly, and cost-effective biotechnological approach for enhancing maize seedling growth in low-fertility soils.

Nevertheless, there were several limitations that might need to be addressed in our future work. First, further investigation is necessary to determine the exact mechanism by which *B. cereus* CGMCC 1.60196 promotes crop growth and development. Specifically, it is important to ascertain whether the strain possesses other crop growth promotion traits (such as nitrogen fixation, phosphate solubilization, and siderophore production) or whether it enhances crop growth through close interactions with other microbial species. Second, additional experiments are needed to study the application effects (Effects on soil properties, crop yield, etc.) of the bacterial inoculant on a variety of soil types and crop types to broaden the application scenarios of its commercialized product. Noteworthy, these verification experiments should include comparative studies with existing biofertilizers to delineate the novelty and effectiveness of this bacterial inoculant. Furthermore, it is essential to study the application effects of the bacterial inoculant across various soil types and crop varieties to enhance the potential commercial product’s application scenarios.

## Data Availability

The original contributions presented in the study are included in the article/supplementary material, further inquiries can be directed to the corresponding author.
